# Exploring Age-Related Changes in Dynamical Non-Stationarity in Electroencephalographic Signals during Early Adolescence

**DOI:** 10.1371/journal.pone.0057217

**Published:** 2013-03-14

**Authors:** Vasily A. Vakorin, Anthony R. McIntosh, Bratislav Mišić, Olga Krakovska, Catherine Poulsen, Kristina Martinu, Tomáš Paus

**Affiliations:** 1 Rotman Research Institute, Baycrest Centre, Toronto, Ontario, Canada; 2 Department of Psychology, University of Toronto, Toronto, Ontario, Canada; 3 Department of Chemistry, York University, Toronto, Ontario, Canada; 4 Montreal Neurological Institute, McGill University, Montreal, Quebec, Canada; 5 Centre de Recherche, Institut Universitaire de Gériatrie de Montréal, Montreal, Quebec, Canada; Hangzhou Normal University, China

## Abstract

Dynamics of brain signals such as electroencephalogram (EEG) can be characterized as a sequence of quasi-stable patterns. Such patterns in the brain signals can be associated with coordinated neural oscillations, which can be modeled by non-linear systems. Further, these patterns can be quantified through dynamical non-stationarity based on detection of qualitative changes in the state of the systems underlying the observed brain signals. This study explored age-related changes in dynamical non-stationarity of the brain signals recorded at rest, longitudinally with 128-channel EEG during early adolescence (10 to 13 years of age, 56 participants). Dynamical non-stationarity was analyzed based on segmentation of the time series with subsequent grouping of the segments into clusters with similar dynamics. Age-related changes in dynamical non-stationarity were described in terms of the number of stationary states and the duration of the stationary segments. We found that the EEG signal became more non-stationary with age. Specifically, the number of states increased whereas the mean duration of the stationary segment decreased with age. These two effects had global and parieto-occipital distribution, respectively, with the later effect being most dominant in the alpha (around 10 Hz) frequency band.

## Introduction

Brain signals recorded, for example, with electroencephalogram (EEG) or magnetoencephalogram (MEG), are naturally variable, and this variability can be characterized in terms of metastability [1]. Specifically, metastability refers to the brain's ability to deviate from one stable state to another, remaining for an extended period of time. Patterns of short oscillatory sequences of neuronal ensembles can be interrupted by periods of stochastic activity [2], [3]. The oscillatory sequences are believed to be a result of the integration of distributed neuronal ensembles, producing coordinated neural oscillations, under the assumption that intrinsic differences in the neuronal activity between the functional modules are sufficiently large to perform their own specific operations [4].

The theory of stochastic processes distinguishes two types of stationarity: strong and weak. The process is called strictly or strongly stationary if all its joint distributions do not change when shifted in time. In signal processing, wherein typical time series are finite, a weaker form of stationarity is routinely employed. A weakly stationary random process has constant mean and variance, and its autocorrelation function depends only on the time lag. Thus, a power spectrum that is constant over time is a manifestation of weak-sense stationarity.

Recent advances in surrogate time series and non-linear analysis showed that neurophysiological signals such as EEG or MEG cannot be fully described by studying their linear properties only [5]. A key assumption in non-linear analysis of EEG/MEG is that there exists a dynamic system underlying the observed time series. From the theoretical point of view, the neural ensembles can be represented by single oscillators [6]. Further, different neural ensembles can be coupled with long-range connections, forming a large-scale network of coupled oscillators. Encouraging results were obtained in modeling the resting state network dynamics wherein the realistic fluctuations in brain signals are considered a result of coupled non-linear systems, in general, with time delays in coupling [7], [8].

We may refer to the variability of a signal, which arises from its non-linear nature, as dynamical non-stationarity - the term used previously in a number of studies on dynamic non-linear properties of the brain signals [9], [10]. Dynamical stationarity may be considered an extension of weak stationarity, which is based on the constancy of linear relations between data points. In contrast to non-stationarity in a stochastic sense, dynamical nonstationarity implies the existence of a non-linear dynamic system associated with an observed signal. We can distinguish two approaches on how to construct a mathematical model of the multistability of observed brain signals. They can be essentially discriminated based on an interplay between the complexity of a model in use and its parameters.

One approach is based on choosing an *a priori* non-linear system. This system should be relatively complex, and able to express nonstationary chaotic behavior for a given set of the parameter values. One example of such a framework is a study, wherein bistability of the alpha brain rhythms, manifested as switching between high- and low-amplitude oscillations, was modeled as arising from a Hopf bifurcation - a local bifurcation in which a fixed point of a dynamical system loses stability [11]. The bursts of two types of neural activity thus corresponded to two noise-induced attractors that exist in a specific region of the parameter space.

The other approach is driven by an idea that in general, a dynamical system underlying the observed time series remains unknown. The goal would be to reasonably approximate the unknown system, often with a set of basis functions. Specifically, the parameters of a model are estimated by fitting a combination of relatively simple functions to different segments of given signals. In this case, dynamical non-stationarity can be understood as external or internal events causing abrupt changes or drifts in system parameters. In the current study, we follow such an approach.

Under this framework, a method based on segmentation of time series and subsequent classification of stationary segments has been proposed to address the issue of non-stationarity of time series [12], [13]. This type of analysis has been applied in several studies of EEG signals. For instance, quantitative characteristics of segmental organization of the brain signals were explored in resting state EEG [14], [15]. Another study reported differences in the duration of stationary EEG segments between patients with mild Alzheimer's disease and healthy subjects [16]. Transitions between dynamical modes have been explored using EEG collected during different sleep stages [17]. Two studies applied an analysis of dynamical non-stationarity to explore temporal structure of epileptogenic EEG [9], [10].

Not much work has been done on exploring age-related changes in non-stationarity either in the context of brain development or aging. A number of studies have characterized, however, age-related changes in variability of brain signals, quantified as sample entropy [18], [19]. For example, the relations between age and variability of EEG signals were analyzed in children (8–15 years) and young adults (20–33 years) performing a face memory task [18]. It was found that the variability of the brain signals increased with age. Similar results were found in other age groups, from one month to five years of age, using EEG collected in response to auditory and visual stimuli [19].

It should be emphasized that sample entropy is a statistic that is closely related to the mean rate of information generated by a non-linear system [20], [21]. In contrast to the presence of linear stochastic effects, it can be used to detect the existence of non-linear deterministic systems underlying the observed signal [22]. In turn, dynamical non-stationarity can reveal the temporal structure of brain signals, identifying the segments characterized by similar dynamics. It would be natural to assume that age-related changes in the variability of brain signals can be characterized by the corresponding changes in dynamical non-stationarity. This hypothesis is tested in this study, which is aimed at exploring the age-related changes in dynamical non-stationarity in terms of the number of stationary states and the duration of stationary segments.

## Materials and Methods

### Data

A total of 65 typically developing adolescents participated in the study at three time points about 18 months apart. Participants who did not have the complete data set collected during all the three visits were excluded from this analysis, leaving a group of 56 adolescents (29 males). The mean and standard deviation of age in each group were as follows: 10
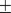
0.4, 11.5
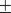
0.4, 13
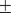
0.4 years old. All participants reported no history of neurological, psychiatric, or developmental disorders. Written informed consent was obtained from the parents, together with assent from the adolescents. The study was conformed to the Helsinki declaration, and approved by the Research Ethics Board of the Montreal Neurological Institute (MNI).

Resting EEG data were acquired using a 128-channel Geodesic Sensor Net and Net Station software, version 3.0.2 (Electrical Geodesics, Inc., Eugene, Oregon, USA). Scalp-electrode impedances were kept between 20 and 60 kOhms. All channels were referenced to Cz during acquisition. EEG recordings were band-pass filtered between 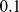 and 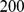 Hz with 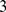 dB attenuation, amplified at a gain of 1000, and digitized with a 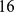-bit A/D converter with a sampling frequency of 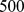 Hz.

The participants were asked to keep their eyes open or closed in 7 alternating 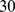 s epochs, with a 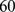 s eyes-closed epoch collected at the beginning and at the end. The data were re-referenced to an average reference and band-pass filtered between 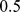 and  Hz. Eight 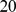 s epochs were extracted from the centre of each segment (5–25 s) to avoid the artifacts associated with opening or closing of the eyes. The mean activity was subtracted from each epoch. Correction for blinks and lateral eye movement was performed using Independent Component Analysis (ICA) with the EEGLAB software [23]. Epochs with remaining artifacts were identified and removed from further analysis, thus leaving, on average, 5 epochs for each visit for the eyes-closed condition, and 4 epochs for the eyes-open condition. For more details on data collection and pre-processing see [24].

### Non-stationarity

Many studies have emphasized the non-stationarity nature of electrical/magnetic brain signals [4], [15], [25]. Typically, it is assumed that the observed EEG or MEG signals are piecewise processes composed of several quasi-stationary components, which in turn reflect the dynamic repertoire of coupled neural ensembles. Our aim is to determine the periods of quasi-stationary dynamics and quantify both their duration and frequency. One way to address this question is through segmentation of nonstationary time series and subsequent classification of quasistationary segments [12], [13].

For segmentation, a given time series 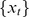, is first divided into 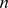 relatively short segments 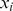, 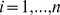, possibly overlapping in time. Then, the difference between the segments in the properties of their dynamics are pairwise computed, producing a matrix 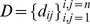 of distances 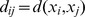, wherein 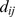 was defined as the Euclidean distance between the vectors of coefficients of a model fitted to each segment of the given time series [14]. According to the Weierstrass approximation theorem, any continuous function on a bounded interval can be approximated by polynomial functions [26]. We define our model in the form of maps [9]:
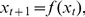
(1)where the function 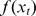 itself is defined in the form of polynomials of some order 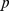 with the coefficients 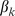, 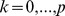:
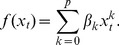
(2)


The distance between two segments 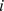 and 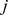 is then defined as
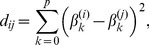
(3)where 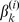 and 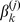 are the corresponding estimated coefficients of the model (2) applied to the segments 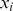 and 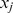, respectively.

After the matrix of distances for a given time series is calculated, the next step (classification) is to unify segments into groups or clusters composed of the segments with similar quasistationary dynamics. In this study, clustering was performed using the affinity propagation algorithm [27]. This is a fast and efficient iterative method that searches for clusters so as to maximize an objective function, called net similarity. The input for the clustering algorithm are the matrix of distances between data points and a parameter that modulates the preferences with which separate data points tend to be unified as one cluster. As a result of clustering, individual segments (or data points in the space of model parameters) are assigned to the identified clusters. It should be noted that, in general, we search for clustering with an unknown number of clusters, which can vary across the participants or channels.

**Figure 1:**
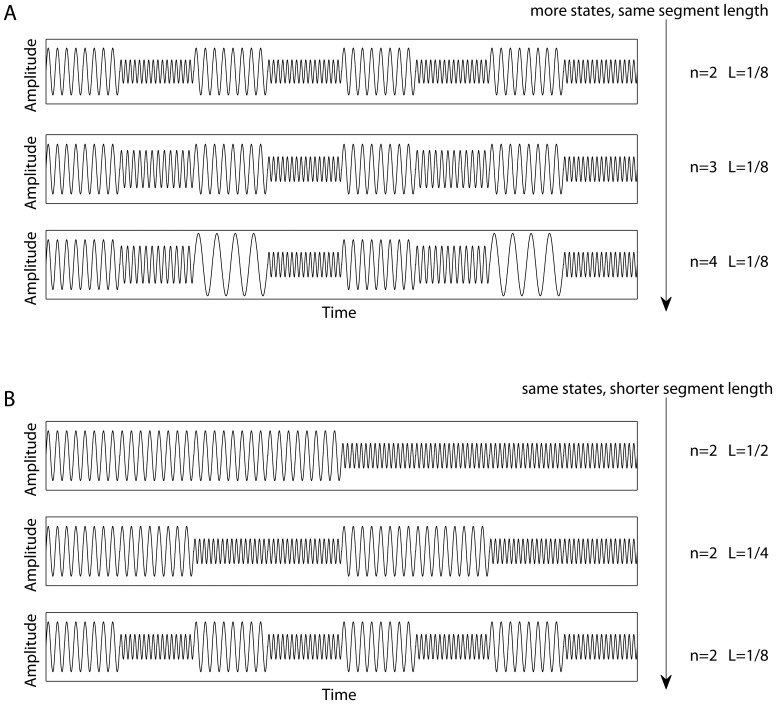
Two mechanisms of non-stationarity. A schematic illustration of two scenarios: (a) n, the number of states, increases, whereas L, their duration, remains the same, and (b) the states get shorter, although the number of states is kept constant.

Finally, the adjacent small segments that belong to the same cluster are glued together to produce a larger segment of quasi-stationary behavior. As a result, a signal can be viewed as a sequence of interlaced segments, each associated with a quasi-stationary state. This signal can be characterized in terms of the number of states (clusters) and the mean segment length. Note that, in general, these two measures are complementary. For example, provided that the number of quasi-stationary states is fixed, higher segment alternation will lead to a smaller mean segment length, as schematically illustrated in Fig. 1b. At the same time, the number of states can be increased without modifying their duration, similar to what is shown in Fig. 1a in a schematic manner.

### Partial least squares

Partial Least Squares (PLS) analysis was used in this study to explore how the two computed measures of non-stationarity (as described above in the previous section) correlate with the age of the participants. PLS is a multivariate technique based on the idea of extracting latent factors that account for most of the variance in the data under investigation. In PLS, the data matrix is decomposed to produce a set of mutually orthogonal factors. Here we give a brief description of the technique, and refer the reader to the original studies for more details [28]–[31].

PLS operates on the entire data structure at once with the data organized in a matrix form. The rows of the data correspond to participants within conditions within groups, whereas the columns correspond to the elements such as voxels in functional magnetic resonance imaging (fMRI), electrodes in EEG, or sensors in MEG. In our case, the elements are represented by EEG electodes. Two forms of PLS analysis can be used: mean-centered and contrast PLS. In the mean-centered PLS, two steps are performed. First, columnwise statistic averages are computed within each condition. Second, the original data matrix is mean-centered with respect to the condition-specific statistic average of the entire column. Mean-centred PLS is a data-driven approach. In the contrast PLS, a design matrix is constructed with *a priori* specified orthogonal contrasts that code for the differences between experimental conditions and groups. At the next step, the brain data are projected into the directions defined by the given contrasts, which yields the data matrix containing the covariance between the design and brain data.

Next, singular value decomposition (SVD) is used to project the mean-centered data matrix or the covariance matrix to a set of orthogonal latent variables (LVs), with decreasing order of magnitude (analogous to principal component analysis). A latent variable consists of three components: (a) a singular value; (b) a vector of the condition loadings (weights within the left singular vector) that represent an underlying contrast; (c) a vector of the electorode loadings (weights within the right singular vector) that represent the optimal relation of the elements to the identified contrast.

Statistical assessment regarding the number of LVs to retain and the importance of individual element weights within a specific LV is based on resampling procedures. The first step is performed using permutation tests, which randomly reassign conditions within subjects. The permutation test assesses the significance of the effect represented in a given LV, by measuring how it is different from random noise. A measure of significance is calculated as the number of times the permuted singular value is higher than the observed singular value. In the second step, the electrode loadings are further tested for stability across participants through bootstrap resampling of participants within conditions. Stability is calculated as the ratio of the loading to the standard error of the generated bootstrap distribution, and is approximately equivalent to a 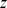-score. For example, the absolute bootstrap ratios higher than 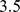 correspond roughly to a 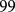
% confidence interval. Electrodes with positive bootstrap ratio values directly support the contrast associated with a given LV. Electrodes with negative bootstrap ratio values also support the underlying contrast, only in a reverse direction.

### Overview of the analysis

The input for an analysis of non-stationarity were the 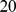 s epochs of resting EEG collected during three visits in the eyes-open and eyes-closed conditions. The analysis was performed, estimating the number of quasi-stationary states and mean segment length for each electrode separately, with subsequent averaging across epochs. Specifically, each of the time series were divided into half-overlapping segments, each containing 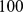 data points. The order of the polynomial model was 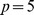. The affinity propagation algorithm was performed in two steps for a given EEG epoch. First, for all the data points to cluster, the input preference parameter was set to the median of all the distances associated with a given matrix of distances between the segments. Then, based on the data points identified as exemplars (centers of the clusters), a second clusterization analysis was performed using the affinity propagation algorithm. At this step, more preferences were given to the exemplars that tended to unify more segments of the initial segmentation. The rationale for this is to avoid the presence of small clusters that unify a few segments, which could result from noisy fluctuations in the model parameters.

Two mean-centered PLS analyses were performed to explore the differences in the number of states and mean segment length across age groups and conditions under the data-driven framework. In addition, four contrast PLS analyses were carried out to explicitly test the significance of the age-related changes and differences in the non-stationarity measures between the two conditions. The significant LVs were determined with 95% confidence. They were visualized in terms of the contrasts they represent between the age groups and/or conditions, and the robustness with which the individual electrodes support the contrasts, data-driven (mean-centered PLS) or given *a priori* (contrast PLS).

## Results

**Figure 2:**
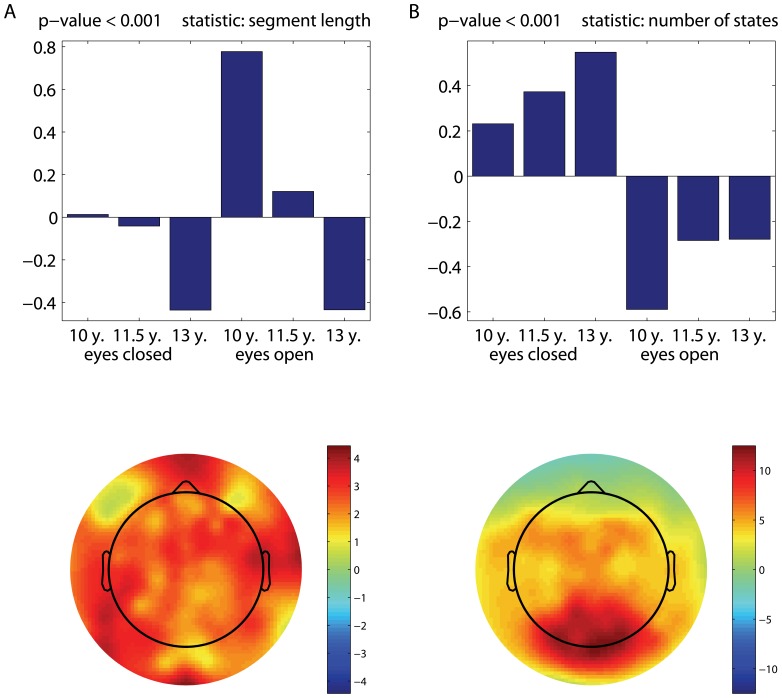
Data-driven contrast between groups and conditions. Age-dependent and condition-specific changes in non-stationarity in terms of: (a) the duration of quasi-stationary states, and (b) the number of states. The patterns of changes represented by the data-driven contrasts from the corresponding mean-centered PLS analyses are shown in the upper parts. The topographic maps (PLS bootstrap ratio values) reflect the spatial distribution of electrode loadings, showing the electrodes' contribution to the identified contrast.

Mean-centred PLS analyses revealed one significant LV for the mean quasi-stationary segment length and also one significant LV for the number of quasi-stationary states. The upper panels in Fig. 2 illustrate the data-driven contrasts (p-value 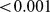 in both cases) underlying the group and condition differences in terms of the mean segment length (Fig. 2a) and the number of states (Fig. 2b). The corresponding overall distributions of the bootstrap ratio values across the electrodes are given in the lower panels of Fig. 2 as the topographic plots. The electrode loadings are mostly positive, indicating that those electrodes directly express the identified statistical trends, as it is shown (without inversion).

These patterns of differences indicate the presence of two effects. First, for both eyes-open and eyes-closed conditions, non-stationarity increases with age. Specifically, as can be seen from Fig. 2, the number of states increases with age, whereas the duration of quasi-stationary segments decreases with age, which implies a more frequent alternation of the states. At the same time, the LV illustrated in Fig. 2b, in contrast to that in Fig. 2a, represents not only the age-related changes in the number of states, but also the contrast between the eyes-open and eyes-closed conditions. Another key difference in the non-stationarity patterns between Fig. 2a and Fig. 2b is how the identified contrasts are expressed by individual electrodes. With regards to the duration of quasi-stationary segments, these effects are distributed across almost all the electrodes, whereas the effects associated with the number of states are robustly expressed only in the parieto-occipital area.

**Figure 3:**
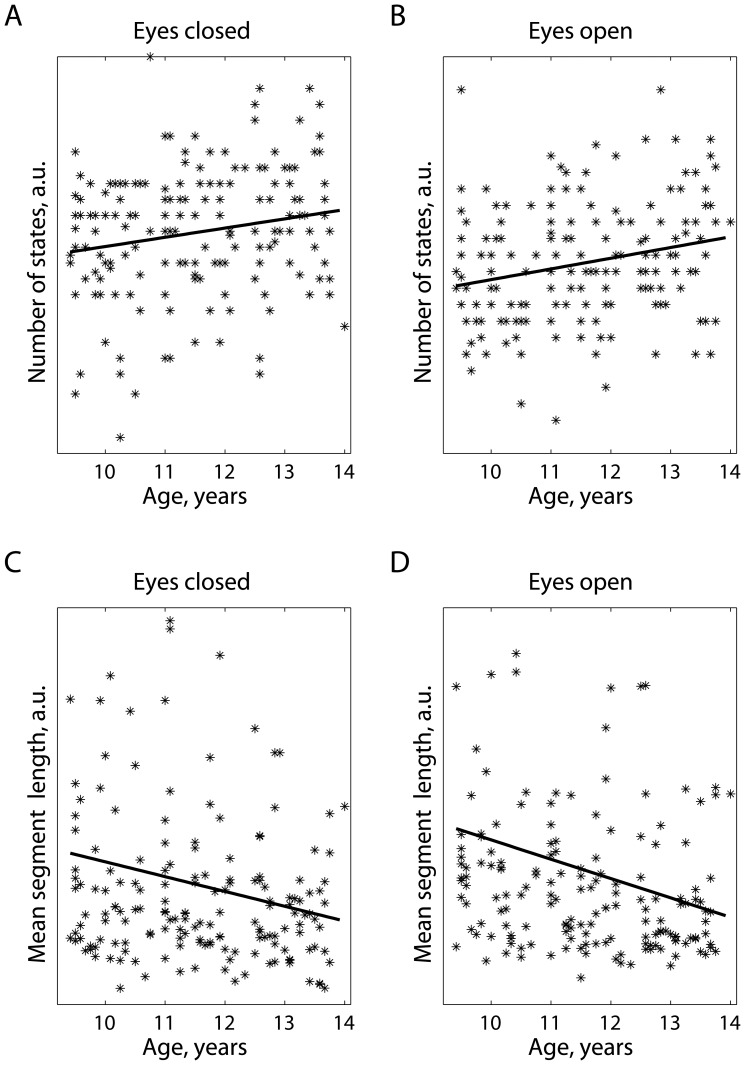
Correlation between age and non-stationarity for one electrode. Relationships between the measures of non-stationarity and age, when no categorization in age groups was done: (a,b) for the number of quasi-stationary states, and (c,d) their mean durations. Each point represents a participant considered at one of three visits. The relationships between non-stationarity and age are illustrated for a specific electrode. Non-stationarity is defined in arbitrary units (a.u.).

To further illustrate the changes in non-stationarity as a function of age, when no categorization was applied in three age groups, we did the following. First, we selected two electrodes: 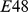 and 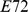 for the mean duration of the quasi-stationary segments and the number of states, respectively. In Fig. 3, four scatter diagrams show the relationships between the two measures of non-stationarity and age, each point representing an adolescent participating during one of the three visits. Each scatter plot was superimposed by a regression line, in all cases with the slope coefficient being statistically different from zero on a 95% confidence interval.

**Figure 4:**
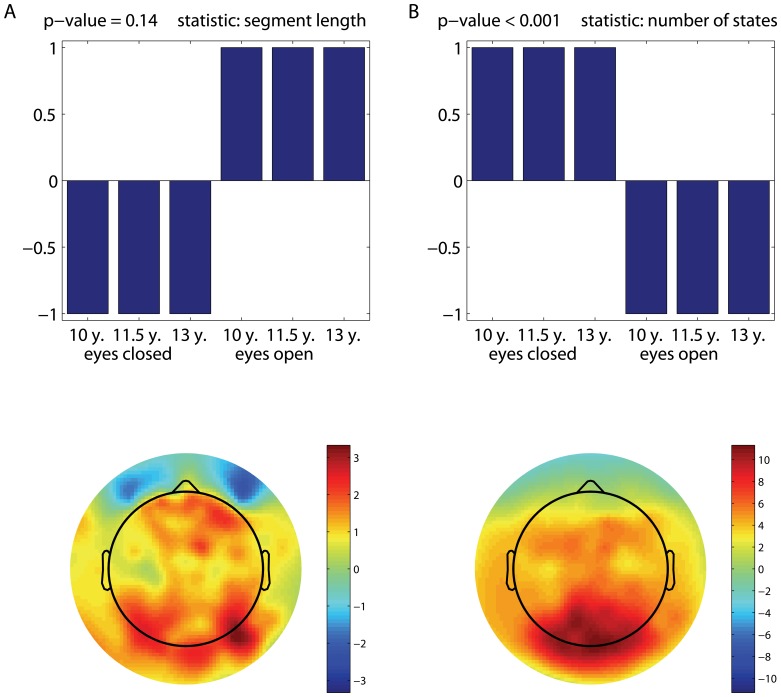
Condition effects (modeled). The “eyes open” versus “eyes closed” hypothesis tested with contrast PLS analyses to track changes in non-stationarity in terms of: (a) the mean quasi-stationary segment length, and (b) the number of quasi-stationary states. Similar to Fig. 2, the topographic maps represent the spatial distribution of the electrodes' contribution to the contrast specified *a priori*.

**Figure 5:**
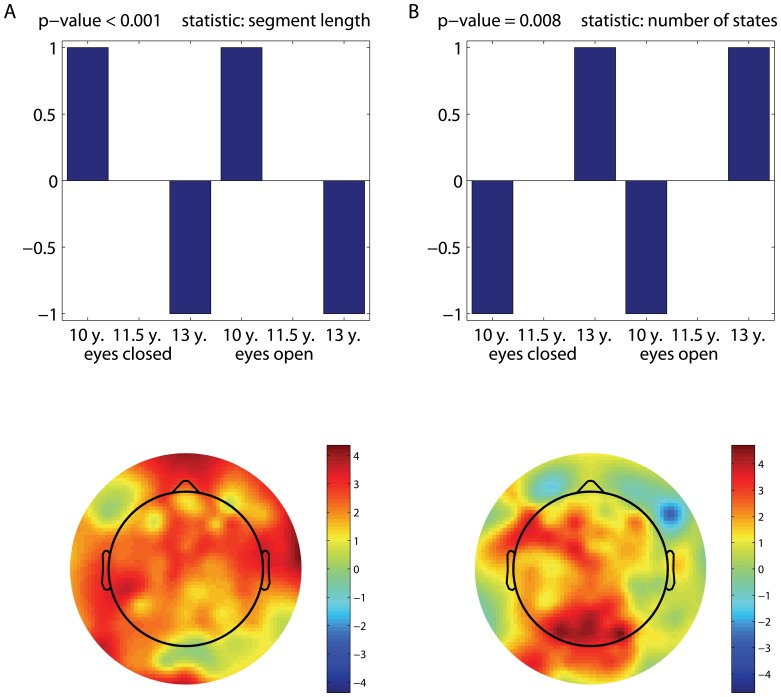
Age effects (modeled). A developmental pattern of changes in non-stationarity, tested with contrast PLS analyses in terms of: (a) the mean stationary segment length, and (b) the number of states. Similar to Fig. 2, the topographic maps reflect the spatial distribution of the LV bootstrap ratio values, revealing the electrodes' contribution to the *a priori* given contrast.

It should be noted that the contrasts shown in Fig. 2 seem to combine both the age-related and condition effects. Note that they were obtained under the data-driven framework (mean-centered PLS). To better clarify these effects, we tested them with two separate contrast PLS analyses, which is a modeling approach. Fig. 4 illustrates the LVs associated with the eyes-open versus eyes-closed contrast, whereas the age-related changes in non-stationarity are illustrated in Fig. 5. Except for the eyes-closed versus eyes-open contrast for the PLS analysis of changes in the duration of quasi-stationary segments (Fig. 4a), which was only approaching significance, the LVs are found to be significant with 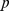-values close to zero. The corresponding p-values are specified at the top of each upper panel in Fig. 4 and 5.

The brain signals collected in the eyes-closed condition contain more quasi-stationary states than those acquired in the eyes-open condition. These effects are captured mostly by the electrodes in the parieto-occipital area (Fig. 4b). Essentially the same electrodes express the effects attributed to an increase in the number of states as a function of age (Fig. 5b). Similar to Fig. 2a, the effects associated with a decrease in the duration of quasi-stationary segments are observed across almost all the electrodes, as shown in Fig. 5a.

**Figure 6:**
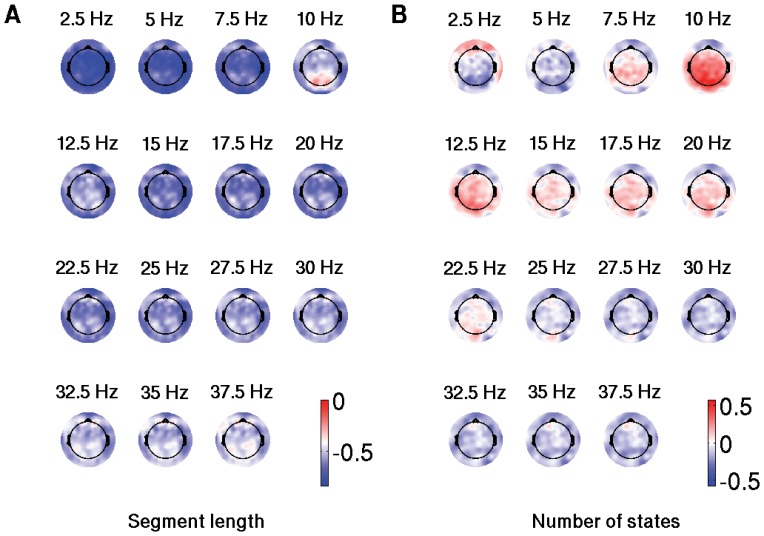
Correlation between spectral power and non-stationarity. Distribution of correlations between relative spectral power and a measure of non-stationarity: (a) mean quasi-stationary segment length and (b) number of quasi-stationary states.

Finally, we calculated the partial correlations between the two measures of non-stationarity and relative spectral power, estimated as described in a previous study that used the same data [24]. Each measure of non-stationarity (the number of states and their duration) was correlated with the relative spectral power, while controlling the other measure (mean duration of states and the number of states, respectively). It was performed on an electrode-by-electrode basis, across subjects, merging groups and conditions. The topographic maps representing the distributions of these correlations across the electrodes are given in Fig. 6. The mean segment length was negatively correlated with the spectral power most strongly at lower frequencies, which was expressed across all the electrodes (Fig. 6a). At the same time, the number of quasi-stationary states was positively correlated with alpha activity (10 Hz). These effects were localized and centered around the parieto-occipital electrodes, as shown in Fig. 6b. It would be worth mentioning that the results in Fig. 6 remained essentially the same when we separated the eyes open and eyes closed conditions (not shown).

## Discussion

This study explored dynamical non-stationarity of resting EEG data collected in the eyes-open and eyes-closed conditions, using age as a perturbation factor. It should be emphasized that we did not study the variability of statistical properties associated with stochastic variables, such as the mean or variance (*i.e.* statistical non-stationarity as defined in the theory of stochastic processes), but rather focused on changes in the parameters of a model, in general non-linear, underlying the observed brain signals (*i.e.* dynamical non-stationarity).

There is a long tradition of defining EEG brain states, which is based on the analysis of the spatial configuration of scalp electric fields [32]. A typical analysis is based on the topography maps defined as a distribution of signal amplitudes at a given moment of time. It can be viewed under the framework of statistical non-stationarity. For example, a non-stationarity analysis was performed to explore continuous sequences of brain electric field maps of resting EEG obtained from a database of participants between the ages of 6 and 80 years [33]. A clustering algorithm was designed to identify four classes of microstate topography and assign each topography map to one of these classes. It was found that the mean microstate duration decreased with age.

In contrast to that study, we characterize the variability of EEG signals under the framework of dynamical non-stationarity [12], [14]. Specifically, dynamical non-stationarity was analyzed through segmentation of individual time series (electrode measurements) with subsequent classification into classes, each associated with a putative brain state. Specifically, a time series was divided into relatively small segments. Then, each segment was fitted to a non-linear model, approximated by polynomial functions, and the parameters describing the dynamics of brain activity on this segment were estimated. The segments were unified into classes characterized by distinctive brain dynamics, through the clustering of the segments in the space of model parameters. Each segment was assigned to a specific cluster (class or state), and adjacent segments belonging to the same cluster were connected to produce larger segments of quasi-stationary behavior. The number of states was not specified *a priori*, but was determined in a data-driven way, in constrast to that study, wherein the number of map classes was not allowed to vary across age groups [33]. In summary, dynamical non-stationarity was characterized not only by the mean duration of quasi-stationary states, but also by the number of different states. Then, a multi-variate analysis (PLS), which combined the data from all the electrodes, age groups and conditions, was applied to detect age-related and condition-specific changes in the two measures of dynamical non-stationarity.

We found that the brain signals became more non-stationary (variable) with the increasing age during early adolescence. Specifically, the mean quasi-stationary segment length decreased, whereas the number of different quasi-stationary states increased with age. In addition, the effects attributed to the age-related changes in the duration of quasi-stationary dynamics were expressed across almost all the electrodes, whereas the effects related to an increase in the number of states were localized to the parieto-occipital channels. The latter was the case for both the eyes-open and eyes-closed conditions.

Similar results were found in a study that introduced a method to decompose the total variability of the signals into local entropy attributed to the dynamics of individual brain areas, and distributed entropy that characterizes the signal variability attributed to coordinated brain activity [34]. The authors explored the interplay between two mechanisms that may contribute to brain variability associated with brain development: larger repertoire of the individualized physiological states of separate brain regions, which become more specialized, and increased integration between distributed neuronal populations. It was shown that the latter mechanism was the key factor contributing to the increased variability of brain signals in development. Specifically, developmental changes were characterized by a decrease in the amount of information processed locally, with a peak in the alpha frequency range. This effect was accompanied by an increase in the variability of brain signals processed as a distributed network.

One limitation of this study is that brain states are defined, and their variability characterized, at the level of EEG electrodes. EEG measurements do not directly represent localized brain regions in the vicinity of one electrode. Rather, due to volume conduction, the measured potentials reflect a summed signal from simultaneously active, underlying current sources [35]. When the signal passes the layers of cerebrospinal fluid, dura, scalp, and skull, it becomes filtered and spread out across electrodes. If there are several local brain states with different dynamics, each electrode will potentially pick up the effects from the different local brain states. On the other hand, a change in an *a-priori* unknown mixture of these effects is itself a change in brain state, but now at a higher level.

It should be noted, however, that the statistics describing non-stationarity should not be interpreted in an absolute sense. Thus, our results do not indicate directly that a brain signal recorded from a participant who belongs to a specific age group (under the eyes-open or eyes-closed condition) is characterized by a certain number of states and a specific duration of the quasi-stationary dynamics. Rather, observed non-stationary effects would strongly depend on the time scales at which time series are being considered. They result from the interplay between the characteristic time scales of an underlying process and observation time. In our case, the estimated statistics characterizing non-stationarity depend on the parameters of segmentation and clusterization, such as the initial length of segments, the order of the polynomial model, and the parameter that controls how big clusters would become after clustering. Nevertheless, this study focused on the age-related changes in non-stationarity, rather than the non-stationary structure of brain signals *per se*. Under this context, we report that the results describing the differences in non-stationarity such as the identified contrast between groups/conditions and topography maps, were robust with respect to a wide range of the parameters that specify the segmentation and clusterization techniques we used.

In addition, correlating the spectral characteristics with the two measures of non-stationarity across subjects, we found that higher spectral power can be associated with more variable dynamics. However, there are potentially two different scenarios describing the variability of brain rhythms at different frequencies, each having its own spatial distribution. On the one hand, increased brain activity at lower frequencies can be modeled as a higher alternation of the same brain states, which is schematically shown in Fig. 1b. On the other hand, the neural activity in the alpha range can be described as a localized generator of brain states. This scenario is modeled in Fig. 1a. Thus, our study provides a basis for gaining more insight into the functional reorganization that takes place in healthy development.
